# An Escape From Diabetes

**DOI:** 10.1210/clinem/dgaa401

**Published:** 2020-06-29

**Authors:** Neda Rasouli

**Affiliations:** University of Colorado, School of Medicine, and VA Eastern Colorado Health Care system, Aurora, Colorado

Type 2 diabetes has been considered a chronic, progressive disease with expected worsening of glycemia due to steady decline in β-cell function over time. However, recent studies have suggested that type 2 diabetes could be a reversible condition in specific settings. In this issue, McInnes et al. (1) investigated whether short-term treatment with a combination regimen of 3 glycemia-lowering medications could induce diabetes remission. Subjects with type 2 diabetes of about 3 years in duration and HbA1c <7% treated with 0 to 2 glycemia-lowering medications (N = 154) were randomized to the “induction” regimen versus standard of care in an open-label fashion. The induction regimen included short-term treatment with metformin + dapagliflozin + insulin glargine as well as lifestyle coaching. Upon completion of 12 weeks of treatment, subjects with A1c <7.3% stopped glycemia-lowering medications (100% in the combination group vs ~70% in the standard of care group) and the rate of remission was evaluated at 24 (primary endpoint), 36, and 48 weeks. In this study, remission was defined as HbA1c <6.5% on no glycemia-lowering medications. The triple combination approach did not result in a significantly higher rate of diabetes remission than the standard of care at 24 weeks. However, in an exploratory analysis, combination therapy compared with the standard of care resulted in a higher rate of diabetes remission at subsequent time points and a lower relapse rate. The failure to meet the primary end point of the study could be multifactorial. The majority of subjects in this study were well controlled without significant glucotoxicity at the baseline and did not have significant weight loss throughout the study. In addition, none of the medications used in the combination therapy are proven to improve β-cell function directly.

Previous studies have shown that sudden and profound decrease in food intake associated with significant weight loss (15% or more) by either bariatric surgery or very low-calorie diet resulted in reversal of type 2 diabetes and restoration of normoglycemia (2, 3). These interventions have not only been associated with improved insulin sensitivity after weight loss but also have resulted in improved β-cell function by decreasing ectopic fat accumulation in the pancreas (4). The follow-up studies suggest that diabetes may remain in remission as long as weight regain is avoided (2, 5). Shorter duration of diabetes and higher initial fasting plasma insulin levels suggestive of better β-cell function are predictors for higher chance for remission in response to weight loss. Intensive insulin therapy in the early stages of diabetes has resulted in remission. Treatment of drug-naïve type 2 diabetes with continuous insulin infusion targeting near-normal range of glycemia for 2 weeks resulted in diabetes remission in about half of the patients at 1 year (6). Relief of glucotoxicity improves β-cell function, which could explain restoring normoglycemia after short-term intensive insulin treatment in patients with newly diagnosed diabetes (7).

Although diabetes remission has been defined as achieving glycemia below the diabetic range in the absence of active pharmacologic therapy, the required duration of drug-free normoglycemia has not been defined. Therefore, it is difficult to compare rates of diabetes remission among studies due to inconsistency in the definition of diabetes remission and differences in the cohorts studied. The rate of diabetes remission by the triple combination therapy (~25%) was significantly lower than the remission rate after a very low-calorie diet (~50%) or bariatric surgery (~70%), suggesting that it would be of great value to explore pharmaceutical interventions with direct effects on improving β-cell function.

Although combination therapy failed to induce diabetes remission more than the standard of care regimen in this study (1), it has been recognized as an alternative to the stepwise approach in early stages of type 2 diabetes (8). The initial combination of metformin, pioglitazone, and exenatide has shown significantly better long-term glycemic control than stepwise therapy. Another ongoing study is comparing initial triple combination regimen with metformin, dapagliflozin, and saxagliptin to the conventional stepwise add-on therapy in drug-naïve patients with recent-onset type 2 diabetes (TRIPLE-AXEL Trial).

The dogma of progressive loss of β-cell function in type 2 diabetes has been recently challenged. Type 2 diabetes can be reversed by weight loss as long as it is done early in the course of disease. With an individualized approach, there is an excellent opportunity within the first 5 to 10 years of diagnosis of type 2 diabetes to restore normoglycemia and escape from diabetes, at least temporarily.

Due to underlying pathophysiologic abnormalities and/or genetic predisposition, patients with type 2 diabetes remain at increased risk for hyperglycemia recurrence. Promoting aggressive reduction in calorie and/or carbohydrate intake and weight loss should be the cornerstone of type 2 diabetes management, particularly early in the course of the disease. Pharmaceutical intervention with direct effects on preserving β-cell function should be the focus of future research. Until then, we need to be cautious about transient remission and continue ongoing monitoring for hyperglycemia and diabetes complications. In addition to hyperglycemia, management of type 2 diabetes includes treating cardiovascular risk factors, such as hypertension and dyslipidemia, which might persist during the remission. Therefore, re-evaluation of cardiovascular risk over time is recommended.

The fable of ‘The Tortoise and the Hare’ could be a metaphor for the management of diabetes. Developing strategies to induce prolonged remission in diabetes is the objective; however, it is important not to be lulled into a false sense of security by a transient remission. Diabetes remission is frequently followed by hyperglycemia recurrence that might be overlooked, causing irreversible complications. Continuous counseling on overall wellbeing of patients should be the foundation of diabetes management.

## Additional Information

***Disclosure Summary:*** N.R. has received research funding from Allergan, Novo Nordisk, Boehringer Ingelheim, and Rhythm Pharmaceutical.

***Data Availability:*** Data sharing is not applicable to this article as no datasets were generated or analyzed during the current study.
